# Supplementary figures and images for: Disrupted structure and aberrant function of CHIP mediates the loss of motor and cognitive function in preclinical models of SCAR16
Source: PLoS Genet. 2018 Sep 17;14(9):e1007664. doi: 10.1371/journal.pgen.1007664 (PMC6160236; doi:10.1371/journal.pgen.1007664)

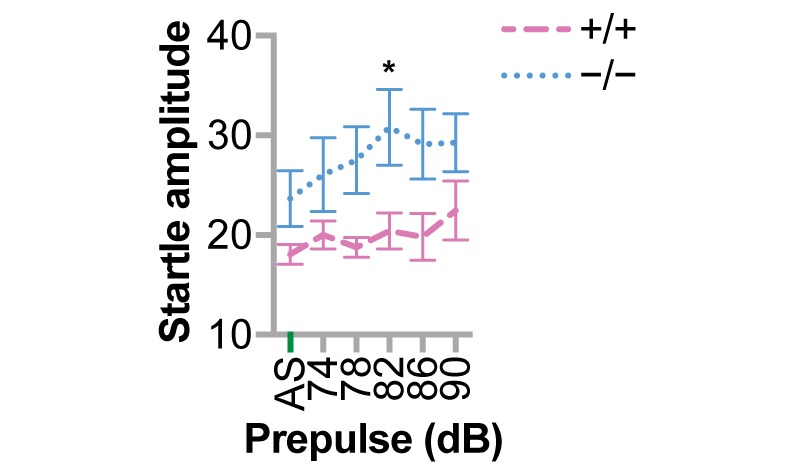

Supplement: S1 Fig — The reaction time of the startle following the acoustic stimulus (AS) in CHIP null mice (−/−, N = 6) and wild-type mice (+/+, N = 10) represented by the mean ± SEM: 2-way ANOVA, p < 0.001 on genotype (main effect), * corresponds to p < 0.05 comparing genotypes via Sidak’s post hoc test. (TIF) [file pgen.1007664.s006.tif]

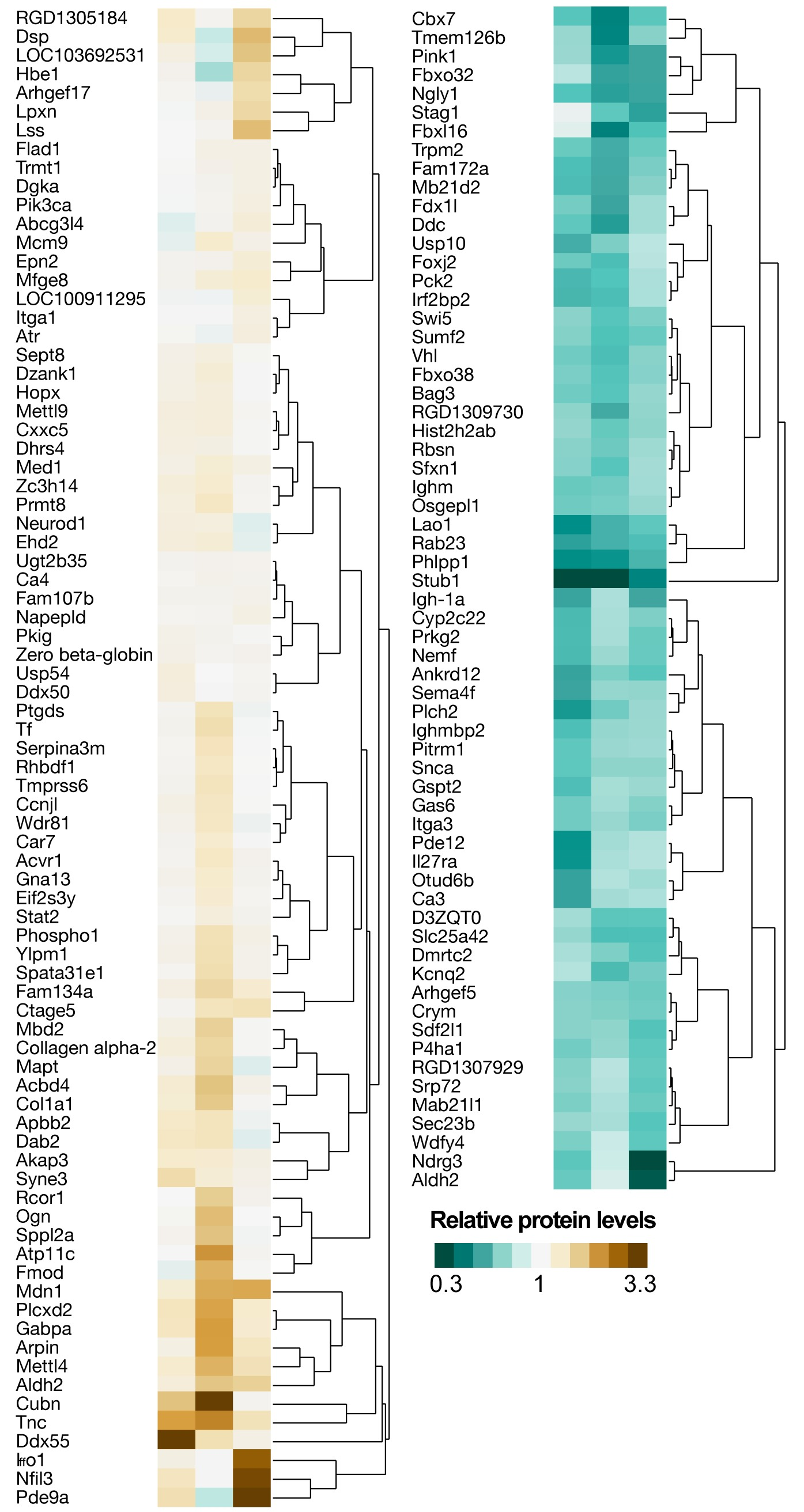

Supplement: S2 Fig — Proteins were clustered using Ward linkage analysis. Each column is the mean of a biological replicate of an M246/M246 cerebellum relative to T246/T246 control cerebellums. The two primary clusters represent proteins that are either increased (left) or decreased (right) in M246/M246 cerebellums as indicated by the color bar. (TIF) [file pgen.1007664.s007.tif]

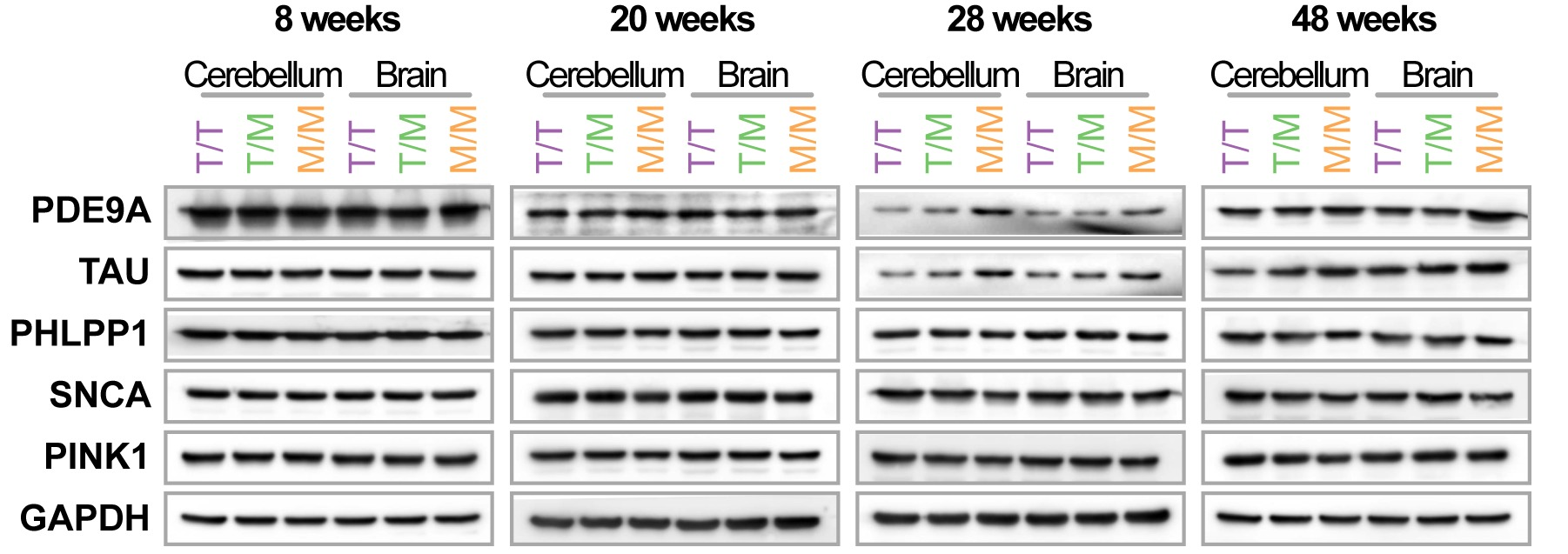

Supplement: S3 Fig — Representative immunoblots of the indicated proteins from either cerebellum or brain extracts isolated from rats with the indicated genotypes and age. (TIF) [file pgen.1007664.s008.tif]

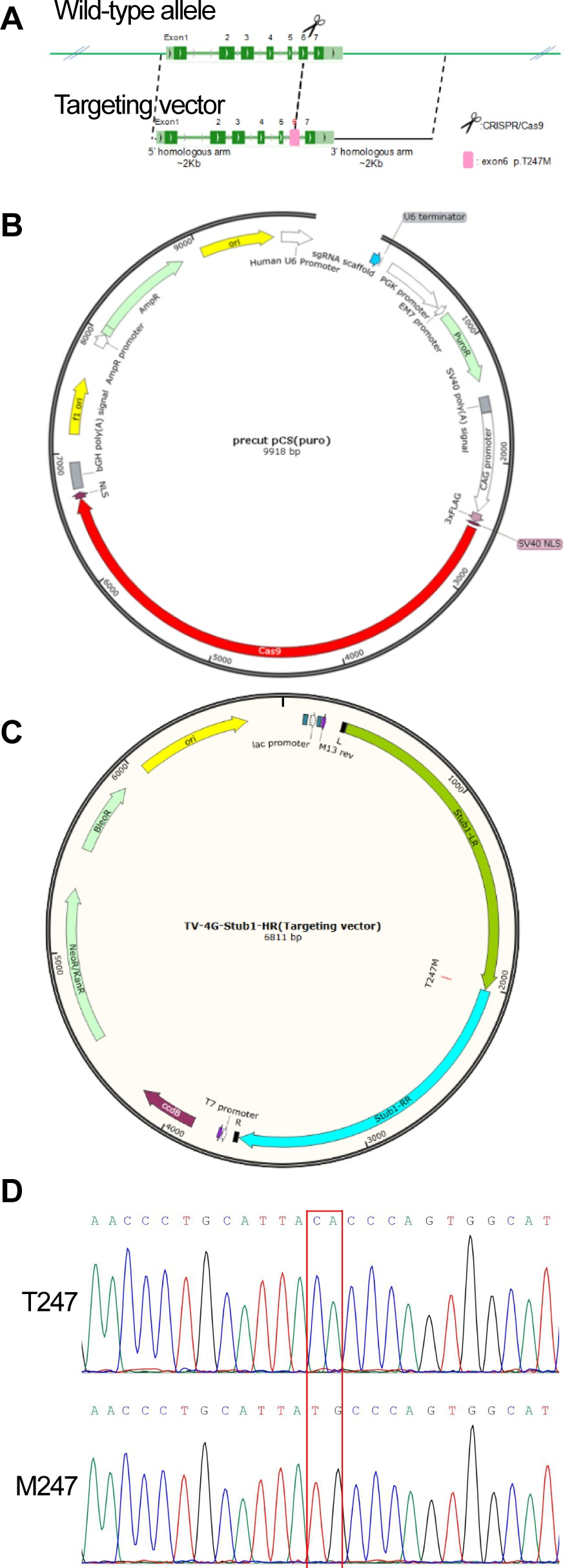

Supplement: S4 Fig — (A) Schematic of targeting vector used for in vivo genome editing, targeting the Cas9 nuclease to exon 6. (B) Map of Cas9 vector. (C) Map of Stub1 T247M targeting vector (rodents have an additional coding exon relative to humans). (D) Sanger sequencing confirmation of the T246M mutation, changing the coding exon from ACA (threonine) to ATG (methionine). (TIF) [file pgen.1007664.s009.tif]
